# Comparison between Different Tricuspid Valve Procedures through Postoperative Inflammation and Myocardial Enzymes

**DOI:** 10.21470/1678-9741-2020-0158

**Published:** 2021

**Authors:** Zuoyong Sun, Zhigang Zhang, Shixiong Wei

**Affiliations:** 1 Department of Cardiothoracic Surgery, Binzhou People’s Hospital, Binzhou, Shandong, People’s Republic of China.; 2 Department of Infectious Diseases, Binzhou People’s Hospital, Binzhou, Shandong, People’s Republic of China.; 3 Department of Cardiovascular Surgery, Chinese PLA General Hospital, Beijing, People’s Republic of China.

**Keywords:** Tricuspid Valve, Sternotomy, Leukocyte Count, Creatine Kinase, Thoracoscopy, Troponin T, Retrospective Studies

## Abstract

**Introduction:**

The thoracoscopic procedure for tricuspid valve (TV) diseases is a minimally invasive method of treatment. This study focuses on comparing the changes in postoperative inflammatory reaction and myocardial injury markers after thoracoscopic and sternotomy/thoracotomy TV procedures.

**Methods:**

We retrospectively analyzed 88 patients (53 males, aged 50.9±16.2 years) with TV diseases (single-valve disease) (72 cases of TV plasty) between January 2018 and April 2019. A total of 56 patients underwent thoracoscopic procedure (50 cases of TV plasty). The leukocyte and C-reactive protein (CRP) levels were monitored as indicators of systemic inflammatory reaction. The lactate dehydrogenase, creatine kinase, creatine kinase myocardial band, aspartate aminotransferase, and troponin-T levels were recorded as markers of myocardial injury.

**Results:**

The CRP and white blood cells levels of patients in the sternotomy approach group were continuously higher than those in patients in the thoracoscopic approach group. And the levels of myocardial enzymes in patients in the thoracoscopic approach group were significantly lower than those in patients in the sternotomy approach group.

**Conclusion:**

Compared with sternotomy/thoracotomy procedures on TV, the thoracoscopic procedure can reduce postoperative myocardial injury significantly and systemic inflammatory reaction to a certain extent. It is technically feasible, safe, effective, and worthy of widespread adoption in clinical practice.

**Table:** 

Abbreviations, acronyms & symbols				
**AST**	**= Aspartate aminotransferase**		**LVEF**	**= Left ventricular ejection fraction**
**BMI**	**= Body mass index**		**MI**	**= Myocardial infarction**
**CK**	**= Creatine kinase**		**N**	**= No statistical value**
**CK-MB**	**= Creatine kinase myocardial band**		**TV**	**= Tricuspid valve**
**CPB**	**= Cardiopulmonary bypass**		**TVP**	**= Tricuspid valve plasty**
**CRP**	**= C-reactive protein**		**TVS**	**= Tricuspid valve surgery**
**I/R**	**= Ischaemia-reperfusion**		**WBC**	**= White blood cells**
**LDH**	**= Lactate dehydrogenase**			

## INTRODUCTION

Accumulating knowledge of the structure, function, and pathology of tricuspid valve (TV) has led to favourable surgical results in TV procedures. Advances in imaging and surgical instruments have allowed surgeons to perform less invasive sternum-sparing TV surgery (TVS). Several kinds of minimally invasive access for TV procedures in adult patients have been popularly introduced into clinical practice. TVS through lower hemisternotomy and right lateral minithoracotomy were safe approaches with very low operative mortality compared to standard median sternotomy^[1,2]^.

Videoscope-assisted cardiac surgery, or VACS, is a platform that provides less incisional trauma but equivalent corrective procedures for cardiac lesion as a conventional open approach. Compared with direct vision in minimal access approach, videoscope may offer smaller incisions, brighter illumination, larger images, and easier recording and broadcasting, whereas it may require more learning curve for eye-hand coordination^[3]^. Here we retrospectively compared and analyzed the characteristics of perioperative inflammatory reaction and myocardial enzyme changes in patients who underwent TVS alone.

## METHODS

### Patients

This was a single-centre, retrospective, and observational study of prospectively collected data from consecutively recruited patients. Written informed consent was preoperatively obtained from each participant and/or their parents or guardians, making them fully informed about the surgical techniques. The decision of surgical approach was made by both doctors and patients. The experiment was approved by our hospital ethics committee.

From January 2018 to April 2019, a total of 88 patients (53 males, aged 50.9±16.2 years, left ventricular ejection fraction [LVEF] 62.6±7.6%) with TV diseases (single-valve disease) came to our hospital for surgical treatment (72 cases of TV plasty [TVP], 83.1%). A total of 56 patients underwent the thoracoscopic procedure (50 cases of TVP) and 32 patients underwent the sternotomy or thoracotomy procedure, and they were divided into the thoracoscopic approach group (n=56) and the sternotomy approach group (n=32), respectively (Table 1).

**Table 1:** Baseline data.

	Thoracoscopic approach group	Sternotomy approach group	*P*-value
Basic characteristics			
Sex (male)	35	18	0.57
Age (years)	49.0±17.9	52.1±17.1	0.878
BMI (kg/m^2^)	22.8±10.0	22.7±14.2	0.704
Height (cm)	158.6±15.4	162.4±10.5	0.318
Weight (kg)	57.3±15.8	60.5±14.9	0.464
Left ventricular ejection fraction (%)	62.3±7.9	63.0±7.3	0.721
Left ventricular end-diastolic diameter (%)	44.7±9.0	45.3±10.2	0.835
Cerebral infarction history	0	0	N
Pleural/pericardial effusion	2	0	0.276
Combined heart disease			
Atrial fibrillation	3	5	0.123
Atrial septal defect	11	7	0.87
Ventricular septal defect	0	3	0.022
Atrial myxoma	5	3	0.986
Infective endocarditis	1	1	0.709
Previous cardiac surgery			
Tricuspid valve procedure	0	3	0.022
Double-valve replacement	2	4	0.125
Mitral valve replacement	9	2	0.166
Aortic valve replacement	1	2	0.288
Correction of congenital heart disease	3	3	0.507

BMI=body mass index; N=no statistical value

### Surgical Procedure

After the induction of general anaesthesia, a 35F left-sided double-lumen endotracheal tube was placed to allow single-lung ventilation. Patients were positioned in supine position with the right hemithorax elevated to 20°. After systemic heparinization, the femoral vessels were cannulated by using Seldinger technique. The setup of a bypass circuit was initiated by positioning a 16-24F catheter in the abdominal aorta through the right femoral artery.

Two small incisions and soft tissue retractors (Aofo Medical Equipment Tech Corporation, People’s Republic of China) were established on the right side of the chest. Port 1 (2-3 cm) was made in the fourth intercostal space on the right anterior axillary line, for the placement of a thoracoscope (Karl Storz Endoskope, Tuttlingen, Germany) and a subsequent chest drainage tube. Port 2 (4-6cm) was made in the fourth intercostal space on a midclavicular line as an entryway for surgical instruments.

Once the previous two ports were secured, a pericardiotomy was performed, and three to four sutures were placed to suspend the pericardium. The right atrium was incised to probe the TV. The diseased TV was resected and replaced with a prosthesis valve on the condition of TV stenosis or repaired if TV regurgitation occurred, at the discretion of the operator. The repair procedure was completed by the insertion of an annuloplasty ring. All annular stitches were exteriorized through the working port and fixed in suture guides. The knots with interrupted sutures were completed extracorporeally and tightened with a knot pusher. The procedure of valve replacement is similar to that of repair. Once the procedure was completed, the right atrium was closed.

### Statistical Analysis

Statistical analysis was performed using IBM Corp. Released 2017, IBM SPSS Statistics for Windows, Version 25.0, Armonk, NY: IBM Corp. Continuous data are presented as the mean with standard deviation. Categorical data are presented as absolute numbers and percentages. Comparisons of patients’ characteristics between groups were performed using the chi-square test for categorical variables and Student’s *t*-test for continuous variables.

### Data Collection

Following the operation, patients were monitored in surgical intensive care units and were transferred to the wards as soon as they were haemodynamically stable. The blood routine, blood biochemical indexes, and coagulation function of each patient were checked on the day of operation and every day after operation routinely. We monitored leukocyte and C-reactive protein (CRP) levels as indicators of systemic inflammatory reaction. Lactate dehydrogenase (LDH), creatine kinase (CK), creatine kinase myocardial band (CK-MB), aspartate aminotransferase (AST), and troponin-T were recorded as markers of myocardial injury.

## RESULTS

### Operational Data

A total of 56 patients underwent thoracoscopic TV procedure and 32 patients underwent sternotomy TV procedure (median sternotomy approach for 26 cases and minithoracotomy approach for six cases). No patient in the thoracoscopic approach group conversed to minithoracotomy or median sternotomy approach. A total of 16 patients underwent TV replacement (thoracoscopic approach group, n=6) and 72 patients underwent TVP (thoracoscopic approach group, n=50). A total of 14 bioprosthetic valves were applied (thoracoscopic approach group, n=6). The overall cardiopulmonary bypass (CPB) time and aortic clamping time were 106.8±54.3 minutes and 80.2±42.7 minutes, respectively (Table 2).

**Table 2:** Operative details.

	Thoracoscopic approach group	Sternotomy approach group	*P*-value
Conversion of surgical approach	0	0	N
Tricuspid valve replacement	6	10	0.06
Tricuspid valve plasty	50	22	
Application of biological valve	6	8	0.094
Cardiopulmonary bypass time (mins)	108.1±52.1	105.0±59.5	0.874
Aortic clamping time (mins)	83.4±47.7	73.8±34.6	0.666
Concomitant operations			
Resection of myxoma	5	3	0.986
Atrial septal defect repair	11	7	0.87
Ventricular septal defect repair	0	2	0.064
Ascending aorta replacement	0	1	0.196
Atrial fibrillation radiofrequency ablation	2	3	0.283

### Postoperative Events

There was only one postoperative death occurrence. The patient underwent double-valve replacement four years before and was admitted to our hospital due to lower limb edema accompanied by wheezing. He underwent redo sternotomy TV replacement. Persistent ventricular fibrillation and low cardiac output occurred on the night after operation. The critical illness and poor cardiac function before operation could have been the cause of death. Two patients of the sternotomy approach group had cerebral infarction and one patient had pulmonary infection. No postoperative adverse event happened in thoracoscopic approach group.

### Comparison of Inflammatory Reaction and Myocardial Enzyme

**Fig. 1:**
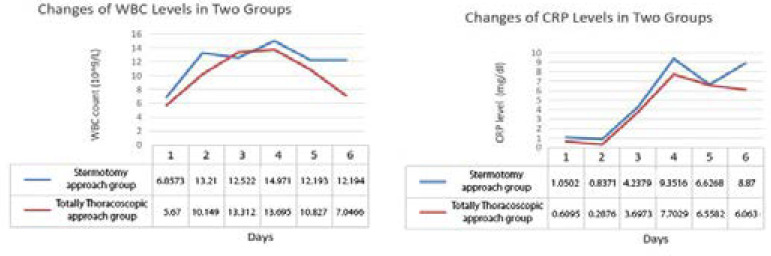
Changes of white blood cells (WBC) and C-reactive protein (CRP) levels in two groups.

**Fig. 2:**
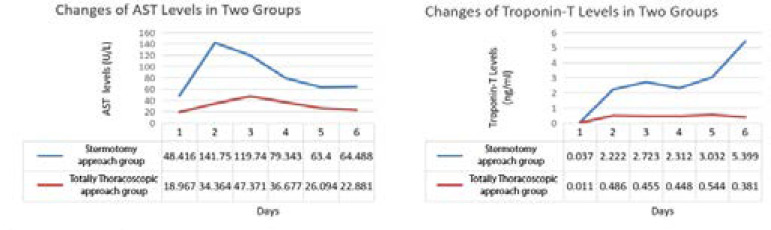
Changes of aspartate aminotransferase (AST) and troponin-T levels in two groups.

**Fig. 3:**
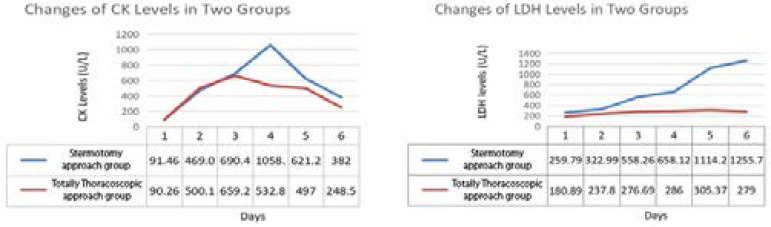
Changes of creatine kinase (CK) and lactate dehydrogenase (LDH) levels in two groups.

**Fig. 4:**
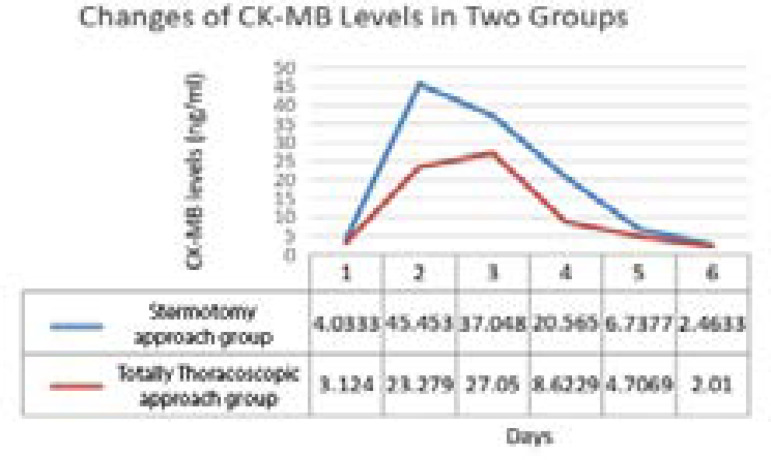
Changes of creatine kinase myocardial band (CK-MB) levels in two groups.

The blood inflammatory index and myocardial enzyme were measured at admission in both groups, and they were continuously monitored on the day of surgery until four days postoperatively (Figure 1). There was no difference in all indexes when patients admitted to hospital (*P*>0.05). The systemic inflammatory reaction of both groups reached its peak on the second day after operation and gradually decreased thereafter. The CRP and white blood cells levels of patients in the sternotomy approach group were continuously higher than those in patients in the thoracoscopic approach group. Besides, the inflammatory reaction of patients in the thoracoscopic approach group is distributed in ladder form, while that of patients in the sternotomy approach group is more fluctuating. In addition, the levels of myocardial enzymes in patients of the thoracoscopic approach group were significantly lower than that in patients of the sternotomy approach group. While the difference of AST, CK, and CK-MB between two groups reached peak value on the next day and the second day after operation, and then tended to be approximately decreased. However, the difference between troponin-T and LDH in both groups showed a continuous increasing trend after surgery (Figures 2 to 4).

## DISCUSSION

Despite improvements in the perioperative management, surgical procedures on the TV still represent a therapeutic challenge. Incidence rate of in-hospital mortality for this demanding patient cohort was reported to be 13-26%^[2]^. The limited outcome associated with isolated TV procedure may be explained by the high rate of redo operations in this cohort, furthermore by the high incidence of postoperative right heart failure and concomitant complications^[4,5]^. Compared with the sternotomy approach, the thoracoscopic approach offers equivalent results without bigger incision wounds for experienced minimally invasive surgeons. Its weaknesses include non-stereo two-dimensional images and less dexterity comparing to the robotic counterpart that can be overcome in the learning curves. Its strengths include lower costs, tactile feedbacks, and less dependency on the assistant, as compared with the robotic approach; the operating surgeon works mostly at the patient’s side, like in conventional operations. Just as general abdominal and thoracic surgeon’s laparoscopic and thoracoscopic operations, there are a variety of selections of scopes, instruments, and accessories, and the operating surgeon must pick the optimally customized specifications of each item^[3]^.

During cardiac surgery different factors, such as the aortic clamp, the extracorporeal circulation, ischaemia-reperfusion (I/R) injury, and endotoxaemia, promote the activation of coagulation pathways, complement factors, and a cellular immune response which can lead to varying degrees of I/R injury or systemic inflammatory response. Higher inflammatory factors have been confirmed to be associated with pulmonary dysfunction, neurological dysfunction, vascular hyporeactivity, lipopolysaccharide-induced myolocation dysfunction, hematological dysfunction, etc. The persistent high level of systemic inflammatory response during perioperative period may even lead to multiple organ failure^[6]^.

We found that the levels of inflammatory factor in patients of the thoracoscopic approach group usually reached peak value on the third day after surgery. And the downward trend is more obvious than that in the sternotomy approach group. On the contrary, the postoperative inflammatory reaction level of patients in the sternotomy approach group was continuously higher than that of patients in the thoracoscopic approach group. After reaching the peak, it still maintained a high level and fluctuated continuously. Among them, the levels of white blood cell of two groups were significantly different on the day after surgery (10.2±2.8 *vs*. 13.2±6.0 10^9/L, *P*<0.05) and the fifth postoperative day (7.0±2.5 *vs*. 12.2±5.4 10^9/L, *P*<0.05).

Myocardial damage is an independent predictor of adverse outcome following cardiac surgery and myocardial protection is one of the key factors to achieve successful outcomes. During CPB, the heart is arrested and protected by cardioplegia. This period is associated with oxygen deprivation and the heart is ischaemic during this time^[8]^. At the end of CPB, the heart is reperfused and cardiac action resumes. These ischaemic and subsequent reperfusion periods cause myocardial injury and even necrosis^[7]^. Myocardial ischaemia causes intracellular calcium accumulation and degradation of the membrane lipids, and oedema during aortic cross-clamping. After removal of the aortic cross-clamp, reperfusion causes oxidative stress depending on the production of reactive oxygen species and reactive nitrogen species. In addition, it has been reported that myocardial I/R induces cardiomyocytic apoptosis^[9-11]^.

Although much less attention is paid to AST in past years, the present study shows that this enzyme still has its clinical relevance. According to literature reported, AST is an independent predictor for early and late mortality after cardiac surgery. It can be used as a screening method for myocardial injury and, therefore, it helps in early detection and treatment of the adverse effects of this serious complication. Although it is not a specific indicator for cardiac damage, it can reflect ischemic effects on other organs (such as the liver and skeletal muscles) as an indirect sign of depressed cardiac function. Another advantage of AST compared with other enzymes is the relatively low cost^[12]^.Our study found that there was a significant difference in postoperative AST levels between two groups immediately after surgery. AST levels on the second, third, and fourth day after operation were statistically different (36.7±18.7 *vs*. 71.3±17.6, 26.1±16.2 *vs.* 54.5±18.9, 22.9±8.8 *vs.* 3.5±14.9 U/L, *P*<0.05). The peak value of patients in the sternotomy approach group appeared on the next day after operation, while that of patients in the thoracoscopic approach group appeared later, reaching its peak in two days after operation. The AST levels of both groups tended to decrease and reached a stable level three days after operation.

Troponins are very specific markers for myocardial infarction (MI) and, currently, the measurement of cardiac troponin-T is the gold standard in laboratory diagnosis of MI, of which the majority are bound to myofilaments and so they are mainly released from necrotic cardiac myocytes. As cardiac troponin increases begin 2-4 hours after acute MI, it is difficult to diagnose acute MI early by measuring it. Early diagnosis of acute MI and early intervention improve the prognosis^[13-16]^. The troponin-T levels of the two groups of patients began to show significant differences the day after surgery. The troponin-T level of patients in the sternotomy approach group showed a continuously fluctuating upward trend, while patients in the thoracoscopic approach group continuously expressed it at a lower level. The troponin-T level of the two groups of patients continued to have statistical difference from the day after surgery (0.5±0.4 *vs*. 2.5±0.9, 0.5±0.5 *vs*. 2.2±0.9, 0.5±0.6 *vs*. 2.7±0.3, 0.4±0.3 *vs*. 3.9±1.5 ng/ml, *P*<0.05).

Previous study demonstrated that CK elevation following cardiac surgery was associated with increased late cardiac death. When patients were categorized according to peak CK elevation, cardiac mortality differed significantly, with a greater mortality observed for patients with high (> 3.0 times normal) and intermediate (1.5-3.0 times normal) CK elevations. CK elevation predicted excessive late cardiac mortality independent of clinical variables, LVEF, the extent and severity of coronary artery disease, coronary lesion morphology, interventional devices, and procedural outcomes^[17]^. The CK levels of both groups showed similar increasing trend on the day of surgery and the day after surgery. However, the CK level of patients in the thoracoscopic approach group reached its peak on the second day after surgery and decreased significantly on the third day after surgery, while the CK peak of patients in the sternotomy approach group appeared later and the peak value was significantly higher than that of patients in the endoscopic group (497.4±247.9 *vs*. 542.6±630.3 U/L, *P*<0.05).

LDH is a widely expressed intracellular enzyme, which reduces pyruvate to lactate during hypoxia. Measurement of plasma LDH levels is rapidly and almost universally available to emergency departments, with increased LDH typically found in hemolysis and in myocardial or skeletal muscle ischemia. Furthermore, increased plasma LDH has been associated with worse outcome and mortality in other conditions such as pneumonia, pancreatitis, hemolysis, thrombosis, and bowel ischemia^[18]^. The changes of LDH level in both groups are very similar to troponin-T, which means that the LDH level in the sternotomy approach group continues to increase, while the LDH level in the thoracoscopic approach group continues to express at a lower level. The LDH levels of the two groups were significantly different on the day after operation (276.7±78.8 *vs*. 496.1±588.0, 286.0±95.4 *vs*. 530.9±506.6, 305.4±122.3 *vs*. 659.6±652.1, 279.0±92.8 *vs*. 614.9 ±595.6, *P*<0.05).

Reports demonstrated that peak CK-MB is the strongest predictor of infarct size, LVEF, and mortality and superior to clinical and angiographic characteristics. The optimal CK-MB cutpoints to discern between small, moderate, and large infarct as measured by cardiac magnetic resonance imaging were defined, and these strongly predicted mortality. Peak CK-MB level was reported to be the superior predictor of histological infarct size in a postmortem study^[19,20]^. The change trend of CK-MB in both groups is different from AST and CK. Although CK-MB levels in both groups showed an increase after operation, they reached its peak value from the next day to the second day after operation, finally showing a downward trend. However, the peak value time of CK-MB in patients in the thoracoscopic approach group was significantly later than that in the sternotomy approach group, and the CK-MB levels of patients in both groups tended to be the same four days after surgery. The CK-MB levels of the two groups were statistically different on the day after surgery (27.1±36.1 *vs*. 34.8±30.5 ng/ml, *P*<0.05).

### Limitations

The limitations of this study are the retrospective study design and the small sample size. At the same time, there is a lack of long-term follow-up for patients. In addition, as this is a single-center study, the results may not be applicable to other cardiac surgery centers.

## CONCLUSION

Compared with sternotomy/thoracotomy procedures on TV, the thoracoscopic procedure can reduce postoperative myocardial injury significantly and systemic inflammatory reaction to a certain extent. It is technically feasible, safe, effective, and worthy of widespread adoption in clinical practice.

**Table:** 

Authors' roles & responsibilities	
SW	Administrative support; substantial contributions to the conception and design of the work; drafting the work; final approval of the version to be published
ZS	Substantial contributions to the conception and design of the work; drafting the work; final approval of the version to be published
ZZ	Substantial contributions to the conception and design of the work; drafting the work; final approval of the version to be published
